# 3-Acetyl-2-methyl-4-(pyridin-3-yl)-1,4-di­hydro­indeno­[1,2-*b*]pyridin-5-one

**DOI:** 10.1107/S1600536813009719

**Published:** 2013-04-13

**Authors:** Imants Bisenieks, Anatoly Mishnev, Imanta Bruvere, Brigita Vigante, Zigmars Andzans

**Affiliations:** a Latvian Institute of Organic Synthesis, 21 Aizkraukles street, Riga LV-1006, Latvia

## Abstract

In the title compound, C_20_H_16_N_2_O_2_, the condensed tricyclic fragment is near to planar, with an r.m.s. deviation of 0.0531 Å. The 1,4-di­hydro­pyridine (1,4-DHP) ring adopts a slightly puckered boat-like conformation. The N and opposite C atoms deviate from the least-squares plane of the four other ring atoms by 0.039 (3) and 0.141 (3) Å, respectively. The C=O group located at the tricyclic fragment is fixed in an *s-trans* orientation, while the second C=O group adopts an *s-cis* orientation with respect to the double bonds of the 1,4-DHP ring. The pyridine ring has a pseudo-axial orientation with respect to the 1,4-DHP ring. The dihedral angle between the tricyclic system and the pyridine ring is 77.3 (3)°. In the crystal, the pyridine N atom accepts a hydrogen bond from the N—H group of the 1,4-DHP ring. The hydrogen bonds link the mol­ecules into infinite *C*(8) chains along the *b-*axis direction.

## Related literature

For general information on the relationship between 1,4-di­hydro­pyridine ring substituents and pharmaceutical effects, see: Christopher *et al.* (2010 ▶); Bisenieks *et al.* (1987 ▶, 1995 ▶); Ivanov *et al.* (1989 ▶). For the synthesis of 1,4-DHP-containing tricyclic derivatives, see: Bisenieks *et al.* (1982 ▶).
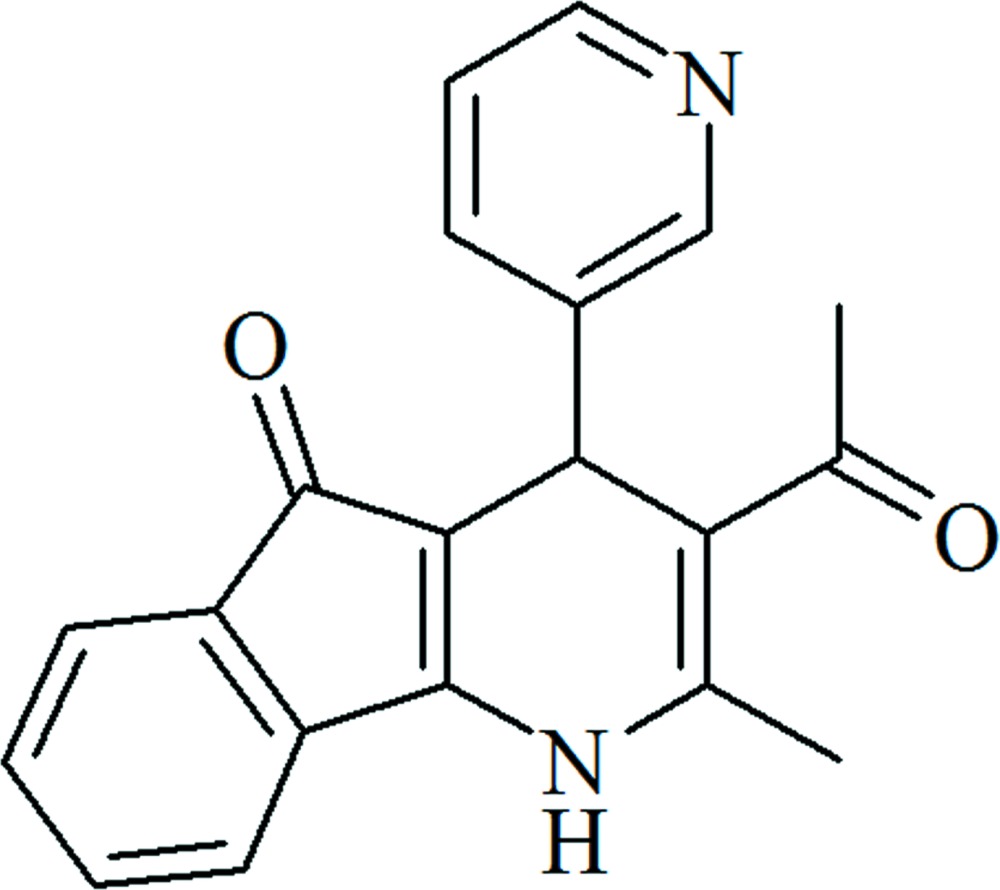


## Experimental

### 

#### Crystal data

C_20_H_16_N_2_O_2_
*M* *_r_* = 316.35
Triclinic, 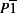

*a* = 8.4361 (3) Å
*b* = 8.8812 (3) Å
*c* = 11.2582 (4) Å
α = 87.692 (2)°
β = 71.022 (2)°
γ = 89.210 (2)°
*V* = 797.00 (5) Å^3^
*Z* = 2
Mo *K*α radiation
μ = 0.09 mm^−1^
*T* = 190 K
0.30 × 0.28 × 0.13 mm

#### Data collection

Nonius KappaCCD diffractometer
5442 measured reflections
3615 independent reflections
2753 reflections with *I* > 2σ(*I*)
*R*_int_ = 0.020

#### Refinement

*R*[*F* ^2^ > 2σ(*F* ^2^)] = 0.047
*wR*(*F* ^2^) = 0.124
*S* = 1.04
3615 reflections
219 parameters
H-atom parameters constrained
Δρ_max_ = 0.23 e Å^−3^
Δρ_min_ = −0.23 e Å^−3^

### 

Data collection: *COLLECT* (Hooft, 1998 ▶); cell refinement: *SCALEPACK* (Otwinowski & Minor, 1997 ▶); data reduction: *DENZO* (Otwinowski & Minor, 1997 ▶) and *SCALEPACK*; program(s) used to solve structure: *SHELXS97* (Sheldrick, 2008 ▶); program(s) used to refine structure: *SHELXL97* (Sheldrick, 2008 ▶); molecular graphics: *ORTEP-3 for Windows* (Farrugia, 2012 ▶); software used to prepare material for publication: *SHELXL97*.

## Supplementary Material

Crystal structure: contains datablock(s) I, global. DOI: 10.1107/S1600536813009719/fy2091sup1.cif

Structure factors: contains datablock(s) I. DOI: 10.1107/S1600536813009719/fy2091Isup2.hkl

Supplementary material file. DOI: 10.1107/S1600536813009719/fy2091Isup3.cml

Additional supplementary materials:  crystallographic information; 3D view; checkCIF report

## Figures and Tables

**Table 1:** Hydrogen-bond geometry (Å, °)

*D*—H⋯*A*	*D*—H	H⋯*A*	*D*⋯*A*	*D*—H⋯*A*
N1—H1*A*⋯N22^i^	0.86	2.06	2.916 (2)	175

Symmetry code: (i) 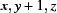.

**Fig. 1.**
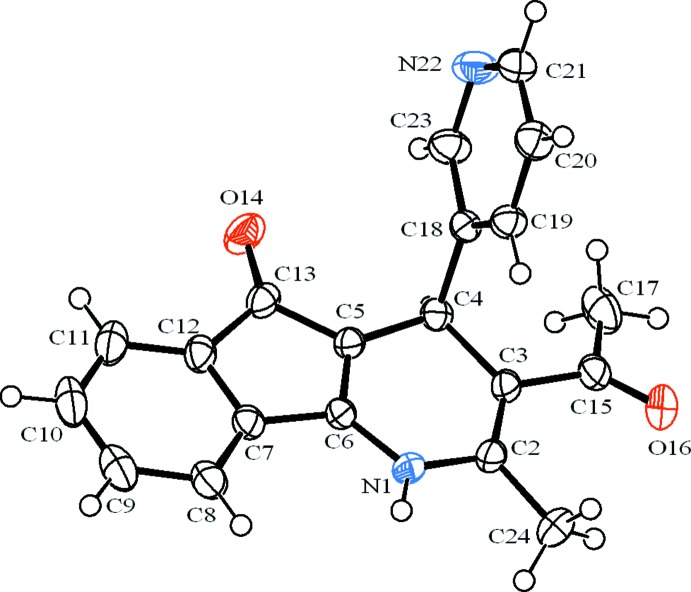
Molecular structure of the title compound showing 50% probability elipsoids.

## supplementary crystallographic information

### Comment

3-Acetyl-2-methyl-4-pyridin-3-yl-1,4-dihydro-indeno[1,2-*b*]pyridin-5-one is a condensed tricyclic 1,4-dihydropyridine derivative containing a conjugated system, with an intense red colour which is common for this type of compounds. In this particular compound, the 1,4-dihydropyridine ring acts as a pharmacophore, and gives potentially useful pharmacological activities. Depending on the particular set of substituents, similar types of 1,4-dihydropyridine derivatives have been indentified as compounds possessing an effect on the accumulation of tau, a microtubule-binding protein, which plays a major role in many neurodegenerative disorders, including Alzheimer's disease (Christopher *et al.*, 2010). It has been reported that similar types of 1,4-dihydropyridine derivatives have anti-cancer and anti-radiation activity (Bisenieks *et al.*, 1995; Ivanov *et al.*, 1989), and also skin regenerating and radioprotection properties (Bisenieks *et al.*, 1987). Some of these types of compounds act as antioxidants (Bisenieks *et al.*, 1982). Fig. 1 shows a view of the crystal structure of the title compound. In the crystal structure the 1,4-dihydropyridine (1,4-DHP) ring adopts a slightly puckered boat conformation. The N1 and C4 atoms deviate from the least-squares plane calculated through the four other ring atoms by 0.039 (3) Å and 0.141 (3) Å, respectively. The carbonyl group C13=O14 is fixed in *trans* orientation with respect to the double bonds of the 1,4-DHP ring while the second carbonyl group C15=O16 assumes *cis* orientation. The condensed tricyclic fragment is planar with an r.m.s. deviation of 0.0531 Å. The pyridine ring has an axial orientation with respect to the 1,4-DHP ring. A dihedral angle between the tricyclic system and the pyridine fragment is 77.3 (3)°. In the crystal, the pyridine nitrogen accepts a hydrogen bond from the N—H group of the 1,4-DHP cycle (Table 1). The hydrogen bonds essembles the molecules in infinite chains, C^1^_1_(8), along the *b* axis.

### Experimental

3-Acetyl-2-methyl-4-pyridin-3-yl-1,4-dihydro-indeno[1,2-*b*]pyridin-5-one was synthesized by the method described by Bisenieks *et al.* (1982). The resulting compound was dissolved in an acetic acid, DMAA and water (2:1:1) mixture while heating at 100 °C. Then the solution was kept at -5 °C until block crystals were obtained.

^1^H-NMR (400 MHz, DMSO-d_6_), δ/p.p.m.: 10.19 (s, 1H, N—H), 8.48–8.49 (m, 1H, py-2-H), 8.34–8.35 (m, 1H, py-6-H), 7.25–7.61 (m, 6H, py-4,5-H and –C_6_H_4_CO–), 4.96 (s, 1H, 4-H), 2.4 (s, 3H, COCH_3_), 2.12 (s, 3H, 2-CH_3_)

### Refinement

The H-atoms were included in the refinement at calculated positions (N—H = 0.86 Å, C—H = 0.93 to 0.98 Å) and refined using a riding-model approximation with *U*_iso_(H)=1.2*U*_eq_(NH,CH) and 1.5*U*_eq_(CH_3_).

### Crystal data

**Table:**

C_20_H_16_N_2_O_2_	*Z* = 2
*M**_r_* = 316.35	*F*(000) = 332
Triclinic, *P*1	*D*_x_ = 1.318 Mg m^−^^3^
Hall symbol: -P 1	Mo *K*α radiation, λ = 0.71073 Å
*a* = 8.4361 (3) Å	Cell parameters from 8654 reflections
*b* = 8.8812 (3) Å	θ = 1.0–27.5°
*c* = 11.2582 (4) Å	µ = 0.09 mm^−^^1^
α = 87.692 (2)°	*T* = 190 K
β = 71.022 (2)°	Plate, red
γ = 89.210 (2)°	0.30 × 0.28 × 0.13 mm
*V* = 797.00 (5) Å^3^	

### Data collection

**Table:**

Nonius KappaCCD diffractometer	2753 reflections with *I* > 2σ(*I*)
Radiation source: fine-focus sealed tube	*R*_int_ = 0.020
Graphite monochromator	θ_max_ = 27.5°, θ_min_ = 2.7°
CCD scans	*h* = −10→10
5442 measured reflections	*k* = −11→10
3615 independent reflections	*l* = −14→14

### Refinement

**Table:**

Refinement on *F*^2^	Primary atom site location: structure-invariant direct methods
Least-squares matrix: full	Secondary atom site location: difference Fourier map
*R*[*F*^2^ > 2σ(*F*^2^)] = 0.047	Hydrogen site location: inferred from neighbouring sites
*wR*(*F*^2^) = 0.124	H-atom parameters constrained
*S* = 1.04	*w* = 1/[σ^2^(*F*_o_^2^) + (0.0583*P*)^2^ + 0.1857*P*] where *P* = (*F*_o_^2^ + 2*F*_c_^2^)/3
3615 reflections	(Δ/σ)_max_ < 0.001
219 parameters	Δρ_max_ = 0.23 e Å^−^^3^
0 restraints	Δρ_min_ = −0.23 e Å^−^^3^

### Special details

**Table:**

Geometry. All e.s.d.'s (except the e.s.d. in the dihedral angle between two l.s. planes) are estimated using the full covariance matrix. The cell e.s.d.'s are taken into account individually in the estimation of e.s.d.'s in distances, angles and torsion angles; correlations between e.s.d.'s in cell parameters are only used when they are defined by crystal symmetry. An approximate (isotropic) treatment of cell e.s.d.'s is used for estimating e.s.d.'s involving l.s. planes.
Refinement. Refinement of *F*^2^ against ALL reflections. The weighted *R*-factor *wR* and goodness of fit *S* are based on *F*^2^, conventional *R*-factors *R* are based on *F*, with *F* set to zero for negative *F*^2^. The threshold expression of *F*^2^ > σ(*F*^2^) is used only for calculating *R*-factors(gt) *etc*. and is not relevant to the choice of reflections for refinement. *R*-factors based on *F*^2^ are statistically about twice as large as those based on *F*, and *R*- factors based on ALL data will be even larger.

### Fractional atomic coordinates and isotropic or equivalent isotropic displacement parameters (Å^2^)

**Table:**

	*x*	*y*	*z*	*U*_iso_*/*U*_eq_	
N1	0.95378 (15)	0.05051 (14)	0.30285 (11)	0.0282 (3)	
H1A	0.9247	0.1425	0.2948	0.034*	
O14	0.77556 (15)	−0.36855 (13)	0.58601 (11)	0.0409 (3)	
C5	0.90043 (17)	−0.18330 (16)	0.41963 (13)	0.0245 (3)	
C6	0.86632 (17)	−0.03576 (16)	0.40434 (13)	0.0243 (3)	
C23	0.9277 (2)	−0.53299 (17)	0.30646 (16)	0.0355 (4)	
H3	0.9676	−0.5603	0.3722	0.043*	
N22	0.85901 (19)	−0.64053 (15)	0.25866 (14)	0.0424 (4)	
C18	0.94286 (16)	−0.38372 (16)	0.26401 (13)	0.0237 (3)	
C3	1.13273 (17)	−0.15786 (17)	0.21807 (13)	0.0258 (3)	
C19	0.87866 (18)	−0.34450 (17)	0.16849 (14)	0.0287 (3)	
H3A	0.8850	−0.2454	0.1373	0.034*	
C15	1.27713 (18)	−0.22251 (19)	0.11903 (15)	0.0336 (4)	
C4	1.02827 (17)	−0.26829 (16)	0.32047 (13)	0.0245 (3)	
H7	1.1027	−0.3218	0.3589	0.029*	
C2	1.09093 (18)	−0.01006 (17)	0.21154 (13)	0.0279 (3)	
C13	0.78546 (18)	−0.24006 (18)	0.53979 (13)	0.0287 (3)	
O16	1.33862 (17)	−0.16140 (15)	0.01533 (11)	0.0523 (4)	
C24	1.1816 (2)	0.1054 (2)	0.11276 (16)	0.0412 (4)	
H11A	1.2993	0.0830	0.0844	0.062*	
H11B	1.1638	0.2036	0.1475	0.062*	
H11C	1.1397	0.1034	0.0431	0.062*	
C20	0.80500 (19)	−0.45370 (19)	0.11972 (15)	0.0346 (4)	
H12	0.7604	−0.4287	0.0560	0.042*	
C7	0.72519 (17)	0.01618 (17)	0.51290 (13)	0.0274 (3)	
C21	0.7984 (2)	−0.59916 (19)	0.16626 (16)	0.0372 (4)	
H14	0.7498	−0.6722	0.1322	0.045*	
C12	0.67731 (18)	−0.10812 (18)	0.59719 (14)	0.0294 (3)	
C8	0.64311 (19)	0.15161 (19)	0.53788 (15)	0.0340 (4)	
H16	0.6747	0.2341	0.4818	0.041*	
C10	0.4653 (2)	0.0398 (2)	0.73495 (16)	0.0434 (4)	
H17	0.3782	0.0497	0.8102	0.052*	
C9	0.5097 (2)	0.1612 (2)	0.65120 (16)	0.0411 (4)	
H18	0.4505	0.2511	0.6699	0.049*	
C17	1.3462 (2)	−0.3722 (2)	0.14648 (18)	0.0454 (5)	
H19A	1.2708	−0.4510	0.1440	0.068*	
H19B	1.3582	−0.3725	0.2284	0.068*	
H19C	1.4536	−0.3885	0.0846	0.068*	
C11	0.54884 (19)	−0.0980 (2)	0.70877 (15)	0.0376 (4)	
H20	0.5183	−0.1803	0.7652	0.045*	

### Atomic displacement parameters (Å^2^)

**Table:**

	*U*^11^	*U*^22^	*U*^33^	*U*^12^	*U*^13^	*U*^23^
N1	0.0341 (6)	0.0202 (6)	0.0261 (6)	−0.0024 (5)	−0.0044 (5)	0.0028 (5)
O14	0.0472 (7)	0.0363 (7)	0.0346 (6)	−0.0057 (5)	−0.0084 (5)	0.0139 (5)
C5	0.0275 (7)	0.0254 (8)	0.0205 (7)	−0.0041 (6)	−0.0078 (6)	0.0001 (6)
C6	0.0267 (7)	0.0260 (8)	0.0204 (7)	−0.0037 (5)	−0.0077 (6)	−0.0022 (6)
C23	0.0484 (9)	0.0247 (8)	0.0374 (9)	−0.0005 (7)	−0.0197 (7)	0.0021 (7)
N22	0.0566 (9)	0.0241 (7)	0.0477 (9)	−0.0066 (6)	−0.0184 (7)	0.0002 (6)
C18	0.0234 (6)	0.0221 (7)	0.0243 (7)	0.0018 (5)	−0.0058 (5)	−0.0023 (6)
C3	0.0262 (7)	0.0304 (8)	0.0199 (7)	−0.0047 (6)	−0.0057 (6)	−0.0028 (6)
C19	0.0318 (7)	0.0245 (8)	0.0305 (8)	0.0000 (6)	−0.0112 (6)	0.0005 (6)
C15	0.0292 (7)	0.0397 (9)	0.0290 (8)	−0.0078 (6)	−0.0040 (6)	−0.0111 (7)
C4	0.0268 (7)	0.0247 (7)	0.0233 (7)	−0.0004 (5)	−0.0097 (6)	−0.0004 (6)
C2	0.0315 (7)	0.0306 (8)	0.0202 (7)	−0.0073 (6)	−0.0062 (6)	0.0006 (6)
C13	0.0300 (7)	0.0339 (9)	0.0231 (7)	−0.0066 (6)	−0.0101 (6)	0.0031 (6)
O16	0.0564 (8)	0.0545 (8)	0.0305 (7)	−0.0109 (6)	0.0084 (6)	−0.0084 (6)
C24	0.0468 (9)	0.0371 (10)	0.0320 (9)	−0.0098 (8)	−0.0027 (7)	0.0075 (7)
C20	0.0344 (8)	0.0404 (9)	0.0322 (8)	−0.0029 (7)	−0.0149 (7)	−0.0036 (7)
C7	0.0270 (7)	0.0321 (8)	0.0238 (7)	−0.0031 (6)	−0.0088 (6)	−0.0049 (6)
C21	0.0370 (8)	0.0359 (9)	0.0372 (9)	−0.0104 (7)	−0.0088 (7)	−0.0084 (7)
C12	0.0277 (7)	0.0388 (9)	0.0220 (7)	−0.0045 (6)	−0.0080 (6)	−0.0031 (6)
C8	0.0350 (8)	0.0355 (9)	0.0322 (8)	0.0010 (6)	−0.0111 (7)	−0.0091 (7)
C10	0.0294 (8)	0.0696 (13)	0.0284 (9)	0.0008 (8)	−0.0041 (7)	−0.0165 (9)
C9	0.0338 (8)	0.0522 (11)	0.0389 (9)	0.0079 (7)	−0.0119 (7)	−0.0198 (8)
C17	0.0326 (8)	0.0541 (12)	0.0476 (10)	0.0104 (7)	−0.0086 (8)	−0.0216 (9)
C11	0.0313 (8)	0.0558 (11)	0.0234 (8)	−0.0073 (7)	−0.0057 (6)	−0.0018 (7)

### Geometric parameters (Å, º)

**Table:**

N1—C6	1.3541 (18)	C2—C24	1.500 (2)
N1—C2	1.3923 (19)	C13—C12	1.507 (2)
N1—H1A	0.8600	C24—H11A	0.9600
O14—C13	1.2274 (19)	C24—H11B	0.9600
C5—C6	1.354 (2)	C24—H11C	0.9600
C5—C13	1.459 (2)	C20—C21	1.369 (2)
C5—C4	1.497 (2)	C20—H12	0.9300
C6—C7	1.486 (2)	C7—C8	1.372 (2)
C23—N22	1.340 (2)	C7—C12	1.400 (2)
C23—C18	1.384 (2)	C21—H14	0.9300
C23—H3	0.9300	C12—C11	1.372 (2)
N22—C21	1.338 (2)	C8—C9	1.405 (2)
C18—C19	1.3837 (19)	C8—H16	0.9300
C18—C4	1.5312 (18)	C10—C9	1.375 (3)
C3—C2	1.360 (2)	C10—C11	1.398 (3)
C3—C15	1.485 (2)	C10—H17	0.9300
C3—C4	1.529 (2)	C9—H18	0.9300
C19—C20	1.382 (2)	C17—H19A	0.9600
C19—H3A	0.9300	C17—H19B	0.9600
C15—O16	1.220 (2)	C17—H19C	0.9600
C15—C17	1.505 (2)	C11—H20	0.9300
C4—H7	0.9800		
			
C6—N1—C2	119.88 (13)	C2—C24—H11A	109.5
C6—N1—H1A	120.1	C2—C24—H11B	109.5
C2—N1—H1A	120.1	H11A—C24—H11B	109.5
C6—C5—C13	108.51 (13)	C2—C24—H11C	109.5
C6—C5—C4	122.68 (13)	H11A—C24—H11C	109.5
C13—C5—C4	128.66 (13)	H11B—C24—H11C	109.5
C5—C6—N1	123.00 (13)	C21—C20—C19	119.23 (14)
C5—C6—C7	111.18 (13)	C21—C20—H12	120.4
N1—C6—C7	125.81 (13)	C19—C20—H12	120.4
N22—C23—C18	124.02 (15)	C8—C7—C12	121.17 (14)
N22—C23—H3	118.0	C8—C7—C6	132.56 (15)
C18—C23—H3	118.0	C12—C7—C6	106.25 (13)
C21—N22—C23	117.45 (14)	N22—C21—C20	122.67 (14)
C19—C18—C23	117.09 (13)	N22—C21—H14	118.7
C19—C18—C4	121.92 (13)	C20—C21—H14	118.7
C23—C18—C4	120.99 (13)	C11—C12—C7	121.20 (15)
C2—C3—C15	120.83 (13)	C11—C12—C13	130.70 (15)
C2—C3—C4	122.57 (13)	C7—C12—C13	108.08 (13)
C15—C3—C4	116.45 (13)	C7—C8—C9	117.61 (16)
C20—C19—C18	119.51 (14)	C7—C8—H16	121.2
C20—C19—H3A	120.2	C9—C8—H16	121.2
C18—C19—H3A	120.2	C9—C10—C11	121.08 (15)
O16—C15—C3	122.70 (16)	C9—C10—H17	119.5
O16—C15—C17	118.81 (15)	C11—C10—H17	119.5
C3—C15—C17	118.46 (14)	C10—C9—C8	121.05 (16)
C5—C4—C3	109.63 (12)	C10—C9—H18	119.5
C5—C4—C18	110.49 (11)	C8—C9—H18	119.5
C3—C4—C18	110.48 (11)	C15—C17—H19A	109.5
C5—C4—H7	108.7	C15—C17—H19B	109.5
C3—C4—H7	108.7	H19A—C17—H19B	109.5
C18—C4—H7	108.7	C15—C17—H19C	109.5
C3—C2—N1	121.09 (13)	H19A—C17—H19C	109.5
C3—C2—C24	126.54 (14)	H19B—C17—H19C	109.5
N1—C2—C24	112.36 (13)	C12—C11—C10	117.86 (16)
O14—C13—C5	127.98 (14)	C12—C11—H20	121.1
O14—C13—C12	126.06 (14)	C10—C11—H20	121.1
C5—C13—C12	105.96 (13)		

### Hydrogen-bond geometry (Å, º)

**Table:**

*D*—H···*A*	*D*—H	H···*A*	*D*···*A*	*D*—H···*A*
N1—H1*A*···N22^i^	0.86	2.06	2.916 (2)	175

Symmetry code: (i) *x*, *y*+1, *z*.
